# Supplementary material for: A deep learning-based system for automatic detection of emesis with high accuracy in Suncus murinus
Source: Commun Biol. 2025 Feb 10;8:209. doi: 10.1038/s42003-025-07479-0 (PMC11811283; doi:10.1038/s42003-025-07479-0)
Supplement: Supplementary file 2 — Supplementary Information [file 42003_2025_7479_MOESM2_ESM.pdf]

Supplementary Table 1. Confusion matrix for test dataset

		Prediction	
		Emesis frames	Non-emesis frames
Observation	Emesis frames	5160	30
	Non-emesis frames	480	25470

Supplementary Table 2 Comparison of the accuracy of emesis detection in Suncus murinus between AED and ActionFormer

	Motion	RTX	Nicotine	Copper sulphate	Naloxone	U46619	Cyclophosphamide	Exendin-4	Cisplatin
AED	99.42%	100.0%	100%	100%	97.10%	98.97%	96.93%	98.91%	98.41%
ActionFormer	98.27%	97.10%	99.10%	100%	97.10%	95.88%	96.93%	95.65%	96.83%

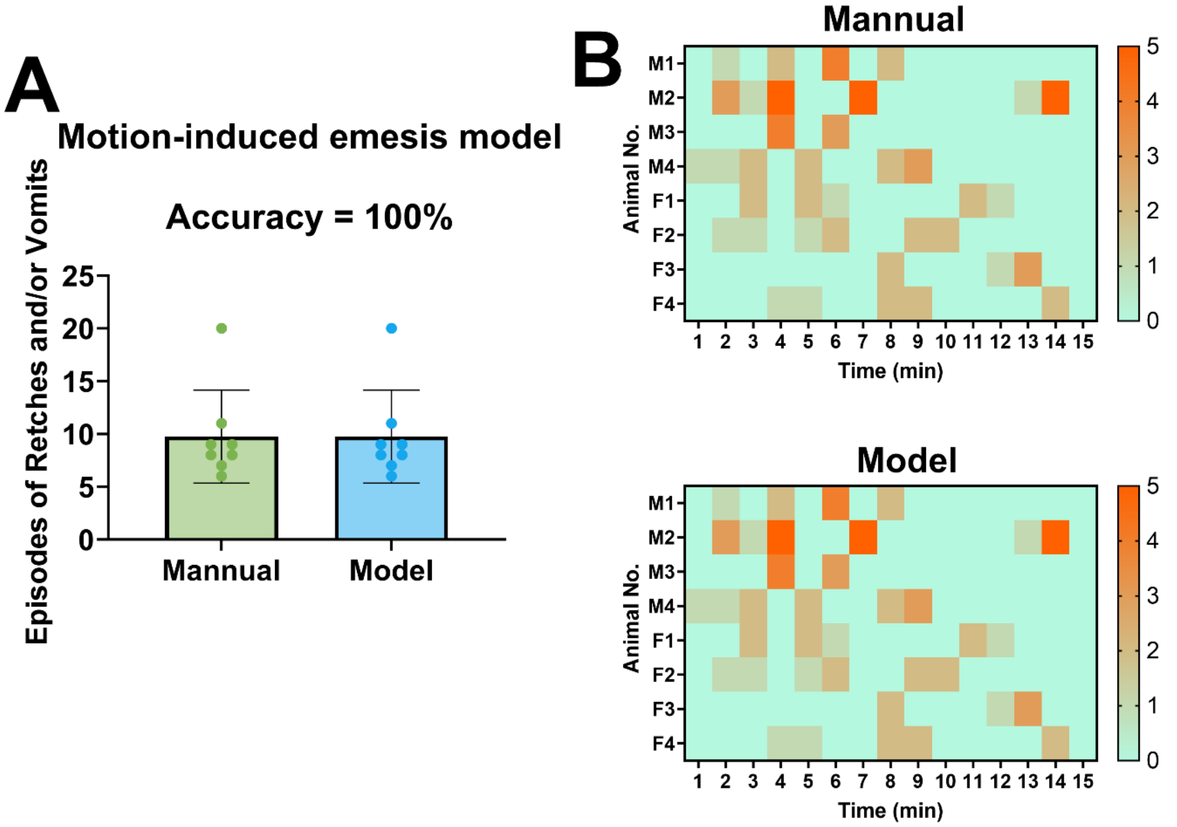

Supplementary Figure 1. Performance of the Automatic Emesis Detection (AED) system in the motion-induced emesis model of *Suncus murinus* in the observation box without bedding. (A) Episodes of retching (R) + vomiting (V) were quantified manually and using the AED system for motion-induced emesis models. (B) Heatmaps showing the latency and number of episodes of R + V per minute for each animal in motion-induced emesis models. Data are presented as the mean ± standard deviation (n = 8 animals). M = male, F = female.
