# Supplementary material for: A deep learning-based system for automatic detection of emesis with high accuracy in Suncus murinus
Source: Commun Biol. 2025 Feb 10;8:209. doi: 10.1038/s42003-025-07479-0 (PMC11811283; doi:10.1038/s42003-025-07479-0)
Supplement: Supplementary file 3 — Description of Additional Supplementary Materials [file 42003_2025_7479_MOESM3_ESM.pdf]

Description of Additional Supplementary Files

File name: Supplementary Videos 1-8

Description: Emetic events that were not detected by the AED tool.

File name: Supplementary Videos 9-14

Description: Non-emetic events that were classified as emetic events by the AED tool.
